# Transmitters and Pathways Mediating Inhibition of Spinal Itch-Signaling Neurons by Scratching and Other Counterstimuli

**DOI:** 10.1371/journal.pone.0022665

**Published:** 2011-07-27

**Authors:** Tasuku Akiyama, Mirela Iodi Carstens, Earl Carstens

**Affiliations:** Department of Neurobiology, Physiology and Behavior, University of California Davis, Davis, California, United States of America; University of Cincinnatti, United States of America

## Abstract

Scratching relieves itch, but the underlying neural mechanisms are poorly understood. We presently investigated a role for the inhibitory neurotransmitters GABA and glycine in scratch-evoked inhibition of spinal itch-signaling neurons in a mouse model of chronic dry skin itch. Superficial dorsal horn neurons ipsilateral to hindpaw dry skin treatment exhibited a high level of spontaneous firing that was significantly attenuated by cutaneous scratching, pinch and noxious heat. Scratch-evoked inhibition was nearly abolished by spinal delivery of the glycine antagonist, strychnine, and was markedly attenuated by respective GABA_A_ and GABA_B_ antagonists bicuculline and saclofen. Scratch-evoked inhibition was also significantly attenuated (but not abolished) by interruption of the upper cervical spinal cord, indicating the involvement of both segmental and suprasegmental circuits that engage glycine- and GABA-mediated inhibition of spinal itch-signaling neurons by noxious counterstimuli.

## Introduction

Itch is often defined as an unpleasant sensation associated with the desire to scratch. Itch provides a warning signal that directs the hand or foot to the itchy skin area so as to remove an insect or other source of irritation by scratching. The protective scratch motion has the added benefit of inhibiting itch sensation. The mechanism by which scratching suppresses itch is not known, but likely involves a central inhibitory mechanism since scratching and other noxious counterstimuli delivered either at [1] or away from the site of pruritogen delivery [2] suppress the itch. It was recently reported that cutaneous scratching inhibits primate spinothalamic tract neuronal activity elicited by the pruritogen, histamine [3]. Since the spinothalamic tract conveys itch as well as pain and temperature sensations [4], the latter results implicate the spinal cord as a critical site for scratch-evoked suppression of itch.

We presently addressed a spinal cord mechanism for scratch-evoked suppression of itch, using a mouse model. Mice exhibit hindlimb scratching directed toward the site of intradermal injection of a variety of pruritogens, including histamine, serotonin and proteases, that also elicit itch sensation in humans [5]–[7]. We used a modification of dry skin model of chronic itch induced by daily skin treatments with acetone/ether/water that results in a significant increase in spontaneous scratching accompanied by increased epidermal thickness, decreased hydration of the stratum corneum and increased transepidermal water loss of the treated skin area [8]. Dry skin treatment of the hindpaw similarly resulted in increased transepidermal water loss and a significant increase in spontaneous paw biting [9]–[10] which is thought to reflect itch [11], with no change in thermal or mechanical sensitivity of the hindpaw [10]. We reasoned that chronic itch from dry hindpaw skin provides tonic input to the lumbar spinal cord that manifests as a high level of spontaneous firing in pruriceptive superficial dorsal horn neurons. We further hypothesized that scratching and other noxious counterstimuli would inhibit the ongoing activity of pruriceptive spinal neurons. We also tested if scratch-evoked inhibition could be attenuated or reversed by antagonizing receptors of glycine and GABA, the two main inhibitory neurotransmitters in the spinal cord. Finally, we addressed the role of segmental and suprasegmental circuits by determining if disruption of the upper cervical spinal cord affected scratch-evoked inhibition of spinal neuronal firing.

## Materials and Methods

Experiments were conducted using 45 ICR mice (Harlan, Oxnard CA) (25–42 g). All work was conducted according to relevant national and international guidelines, under protocol #16420 that was approved by the UC Davis Animal Care and Use Committee.

To induce chronic dry skin on the hindpaw, we followed a previously-reported procedure [10], [12]. Briefly, either one or both hindpaws were wrapped with gauze soaked with a mixture of acetone and diethylether (1∶1) for 15 s, followed immediately by distilled water for 30 sec, twice-daily for 10–12 days. Mice were fitted with a plastic Elizabethan collar (diameter 11 cm) placed around the chest just beneath the forelimbs to prevent any biting or licking of the treated hindpaw(s).

Following the final treatment day, the mouse was anesthetized with sodium pentobarbital (60 mg/kg ip) and prepared for single-unit recording from the lumbar spinal cord as previously detailed [12]. A tungsten microelectrode was driven into the superficial dorsal horn ipsilateral to the dry skin treatment, and a spontaneously firing extracellular action potential was isolated. Unit activity was amplified, digitized and displayed on computer using a Powerlab (AD Instruments, Colorado Springs CO) interface.

As we reported previously [12], the ongoing activity of the isolated neurons hampered the ability to accurately map mechanosensitive receptive fields. Most units were tested for the effect of scratching on ongoing activity. Scratching was accomplished by moving a brush bristle approximating the size of a mouse toenail in a back-and-forth motion across the ventral surface of the hindpaw at a frequency of ∼2 Hz, excursion of ∼5 mm across the ventral hindpaw, and a force of 300 mN for a period of 60 sec. The scratch stimulus covered approximately 1/3 to 1/2 of the length of the hindpaw. Spontaneous neuronal activity was recorded for 60 sec, followed by a 60 sec period of scratching, and lastly by another 60 sec of spontaneous firing. A smaller number of units was similarly tested with non-noxious brushing delivered for 60 sec. We also tested effects of noxious heat (48-56°C; 20 sec duration) and cold (−11°C; 30 sec duration), and in a few cases noxious pinch stimuli (5 sec). Thermal stimuli were delivered from an adapting temperature of 35°C using a computer-controlled Peltier thermode (NT-2A Physitemp, Clifton NJ).

In one set of experiments we sought to determine if the spinal application of antagonists of GABA and glycine receptors affected scratch-evoked inhibition of neuronal activity. A gravity-driven perfusion system allowed artificial cerebrospinal fluid (Krebs: 117 mM NaCl, 3.6 mM KCl, 2.5 mM CaCl_2_, 1.2 mM MgCl_2_, 1.2 mM NaH_2_PO_4_, 25 mM NaHCO_3_ and 11 mM glucose which was equilibrated with 95% O_2_ and 5% CO_2_ at 37°C) to be superfused continually over the exposed lumbosacral spinal cord. Antagonists were delivered by superfusion for a period of 30 sec, followed immediately by ACSF. The effect of scratching was assessed as described above, before and again 1 and 5 min after application of the antagonist. Antagonist concentrations were: strychnine 4 µM, bicuculline 20 µM [13] and saclofen 100 nM, all dissolved in ACSF. Some units were tested with more than one antagonist, since scratch-evoked inhibition was always observed to recover following delivery of each antagonist. In animals receiving bilateral hindpaw dry skin treatment, a second unit was recorded on the opposite side of the spinal cord. In a few animals, a third unit was also recorded.

In 6 mice, the upper cervical spinal cord was exposed by laminectomy to allow superfusion with chilled (0°C) Ringers to provide cold-block of ascending and descending axonal conduction. To verify intraspinal cooling, a small implantable thermocouple with rapid response time (IT-21, Physitemp, Clifton NJ) was inserted into the cervical ventral horn. The thermocouple was connected to a digital thermometer. Following application of ice, the minimum temperature achieved in each experiment was 21°C, a temperature previously reported to greatly reduce neuronal activity [14]. In 9 mice the upper cervical spinal cord was transected surgically. Complete transection was verified by post-mortem visual inspection.

At the conclusion of recordings, an electrolytic lesion was made. The spinal cord was postfixed in 10% buffered formalin and cut in 50 µm frozen sections to identify the lesion sites.7,

The degree of scratch-evoked inhibition was calculated as the mean sum of spikes/60 sec during scratching divided by the mean sum prior to scratching. Unit spike counts during the 60 sec period of scratching, and during the 60 sec epochs before and after the period of stimulation, were compared by paired t-tests. Neuronal firing prior to and during application of brush (60-sec epochs), pinch (5-sec epochs), noxious heat (15-sec epochs) and cold (30 sec epochs) were similarly compared. The correlation between spontaneous firing rate and degree of scratch-evoked inhibition was assessed by Pearson's produce moment correlation (SPSS 9.0, Chicago, IL). To test effects of antagonists on scratch-evoked inhibition, mean spike counts during scratching in the presence of antagonist were compared with those prior to, and 5 min after cessation of antagonist delivery, using paired t-tests. Neural activity during scratching prior to, and during cervical spinal cold block, or following cervical transaction, was similarly analyzed. A p value<0.05 was considered statistically significant.

## Results

### Effects of scratching and other stimuli on spontaneous firing

**Figure 1:**
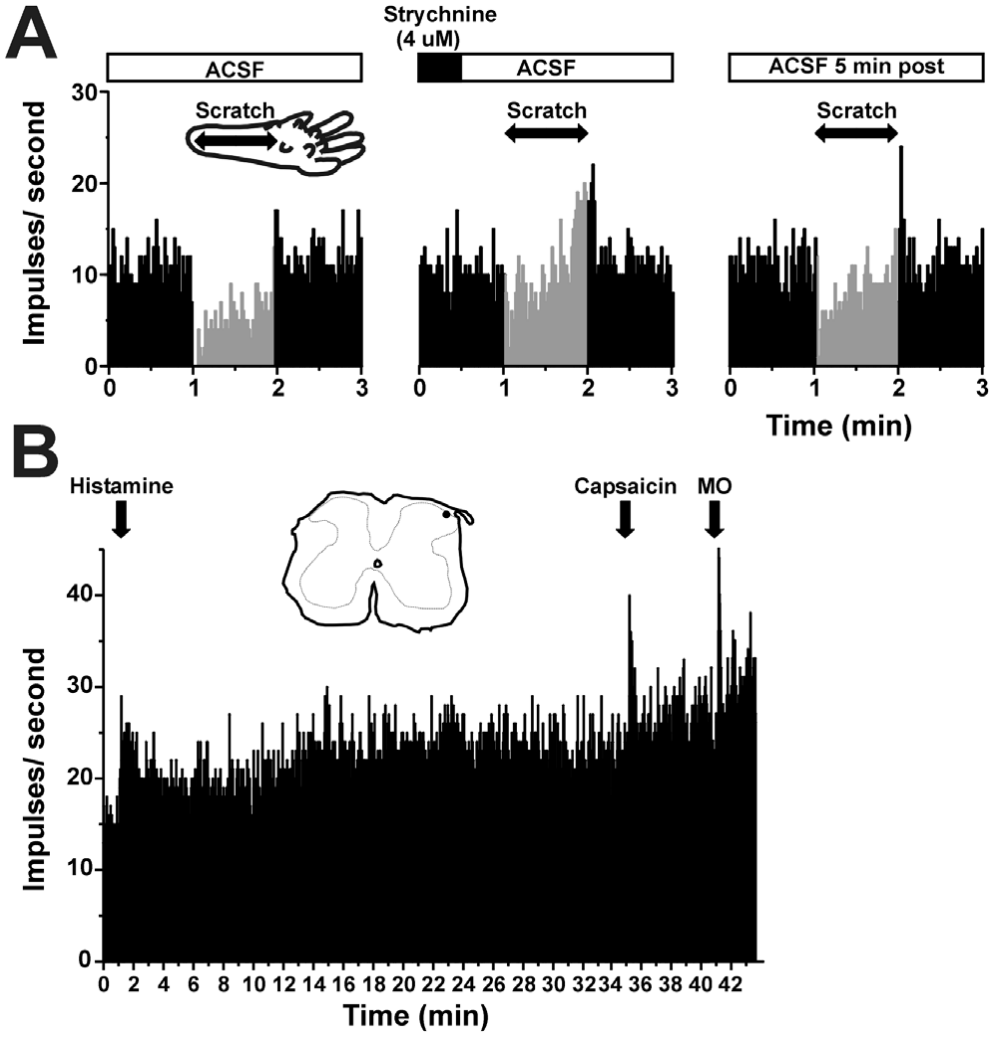
Scratch-evoked inhibition of spinal neuronal firing is blocked by strychnine. A: Shown are peristimulus-time histograms (PSTHs; bins: 1 sec) of a superficial dorsal horn unit's ongoing firing. The gray portion of the PSTH indicates the time that the scratch stimuli were applied. The left-hand PSTH shows that under control conditions of spinal superfusion with artificial cerebrospinal fluid (ACSF; upper bar), repetitive back-andforth scratch movements across the ventral hindpaw markedly depressed firing. The inset shows the 60-sec duration of scratching (arrows) on a drawing of the hindpaw indicating the direction of scratch motions. When strychnine was delivered by spinal superfusion for 30 sec (middle PSTH; black bar above), scratching was much less effective in reducing neuronal firing. The right-hand PSTH shows partial recovery of scratch-evoked inhibition 5 min post-strychnine. B: PSTH showing response of same unit in A to intradermal injection of histamine (arrow; 50 µg/µl). Note the rapid and sustained increase in firing rate. The unit also responded phasically to intradermal injection of capsaicin (35 µg/µl) and topical application of mustard oil (MO). Inset shows a spinal cord section with the histologically recovered recording site (•) in lamina I.

**Figure 2:**
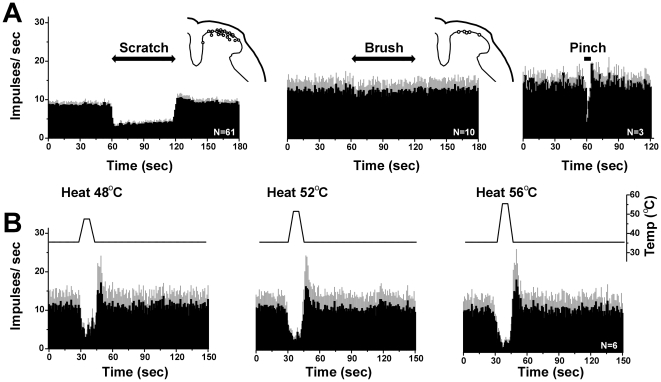
Effects of scratching and other stimuli on spontaneous activity. A: Averaged PSTHs (bins: 1 sec) show, from *left* to *right*, unit responses to scratch (*n* = 61), brush (*n* = 10) and pinch (*n* = 3). Gray error bars: SEM. The 60-sec duration of scratching, but not brush, markedly depressed firing. The 5-sec duration of pinch also markedly depressed firing. B: Averaged PSTHs (bins: 1 sec) show, from *left* to *right*, unit responses to 48°C, 52°C and 56°C heat stimuli (*n* = 6). Gray error bars: SEM. Upper trace shows skin temperature. Heat stimuli markedly depressed firing in a temperature-dependent manner, followed by rebound.

**Figure 3:**
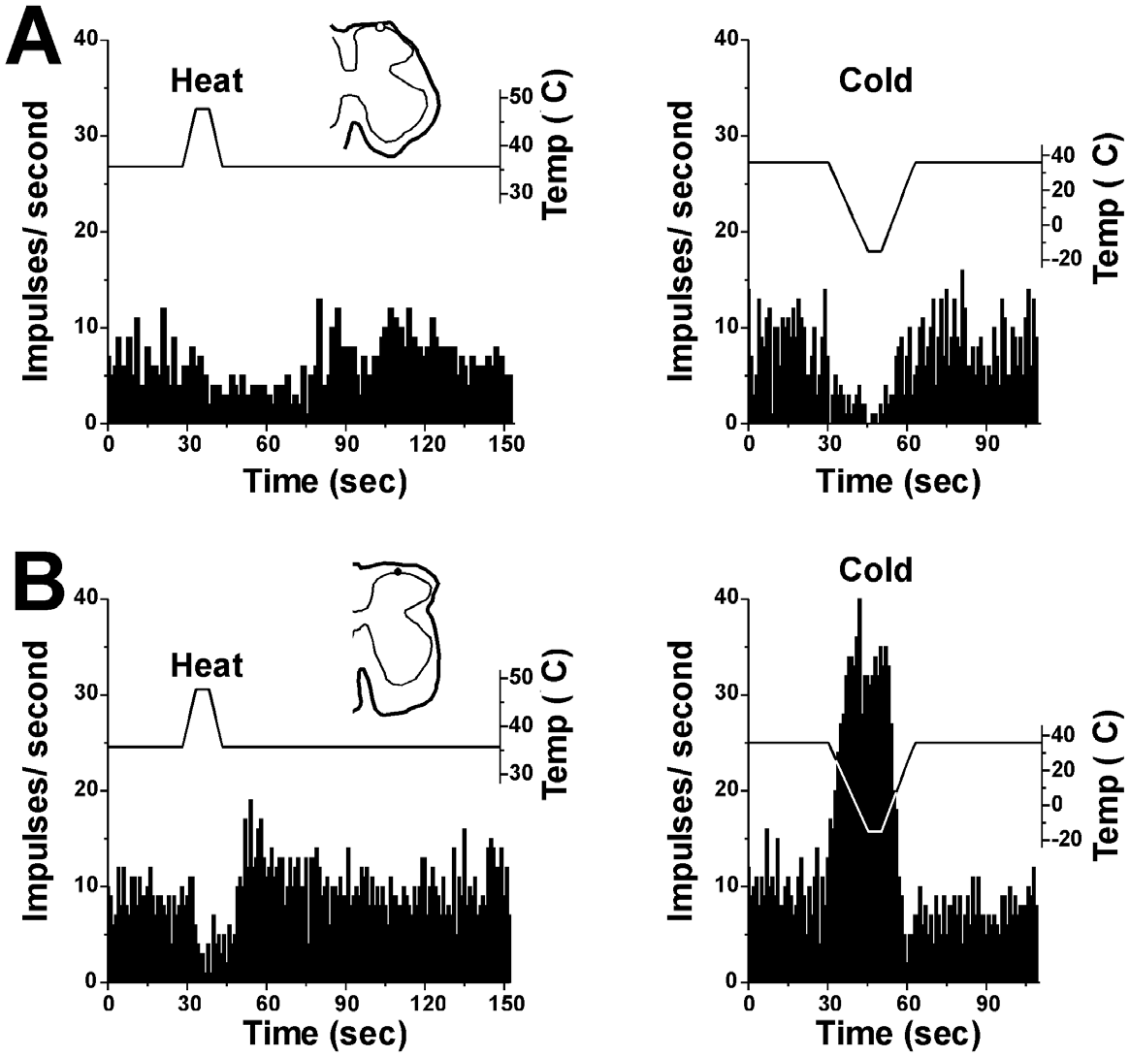
Effects of heat and cooling on spinal neuronal firing. A: PSTHs (bins: 1 sec) showing response of a superficial dorsal horn unit to thermal stimulus. Left top panel shows 48°C heat-evoked depression of firing. Right top panel shows −5°C cold-evoked depression of firing. Bottom graph shows temperature. B: PSTH showing response of different unit in A to thermal stimuli. Left panel: 48°C heat stimulus depressed firing. Right panel shows −5°C cold-evoked enhancement of firing. Traces above PSTHs show temperature.

Recordings were made from 71 units ipsilateral to the dry skin-treated hindpaw. They were located in the superficial dorsal horn at a mean depth of 127.9 µm +/− 9.1 (SEM) below the surface, with most histologically recovered sites in the superficial dorsal horn (Figs. 1B, 2A, 3). All units exhibited spontaneous activity ranging from 0.5–26.1 Hz with a mean of 7.5 Hz +/−0.6 (SEM). The mean level of spontaneous firing was significantly greater (p<0.001) compared to that of 40 superficial dorsal horn units recorded in naïve mice (1.1 +/−0.2 Hz) [15]. Because of the spontaneous activity, it was not possible to accurately map receptive fields in many units. Of 11 units whose mechanical sensitivity was tested, two responded differentially to innocuous (brush) and noxious (pinch) stimuli and were classified as wide dynamic range (WDR), two responded to pinch but not brush and were classified as nociceptive-specific (NS), while 7 did not respond to mechanical stimulation.

Scratching of the dry skin-treated ventral hindpaw skin ipsilateral to the recorded unit resulted in a phasic reduction in ongoing firing by >30% in almost all (56/61) units tested, followed by a rapid recovery to the pre-scratch firing level when scratching ceased. An example is shown in Fig. 1A (left-hand peristimulus-time histogram =  PSTH). This unit, located in lamina I, exhibited increased firing following id injection of histamine, as well as phasic responses to capsaicin and AITC (Fig. 1B), consistent with our prior report [12]. The left-hand PSTH in Fig. 2A shows the mean response of 61 spontaneously active units before, during and after scratching. During scratching, firing was significantly (p<0.001, paired t-test) reduced to a mean of 43.8% of the pre-scratch baseline firing rate, followed by a rapid rebound at the cessation of scratching. In contrast to the inhibitory effect of scratching, innocuous brushing of the dry-skin-treated ventral hindpaw had no significant effect on spontaneous activity of dorsal horn units (Fig. 2A, middle PSTH). There was a significant inverse correlation between the spontaneous firing rate and the degree of scratch-evoked inhibition (r^2^ =  0.16, p<0.001, Pearson's product moment correlation), indicating that scratching was less effective in units with higher spontaneous firing rates.

Noxious heating at each temperature tested also significantly reduced ongoing firing in 6 units (Fig. 2B; p<0.05 for each). The left PSTHs in Fig. 3A, B show two examples in which noxious heating of the ventral hindpaw attenuated ongoing firing. Interestingly, noxious cooling had variable effects, with 1 unit exhibiting a marked reduction (Fig. 3A, right PSTH) and 5 units exhibiting increased firing (Fig. 3B, right PSTH).

**Figure 4:**
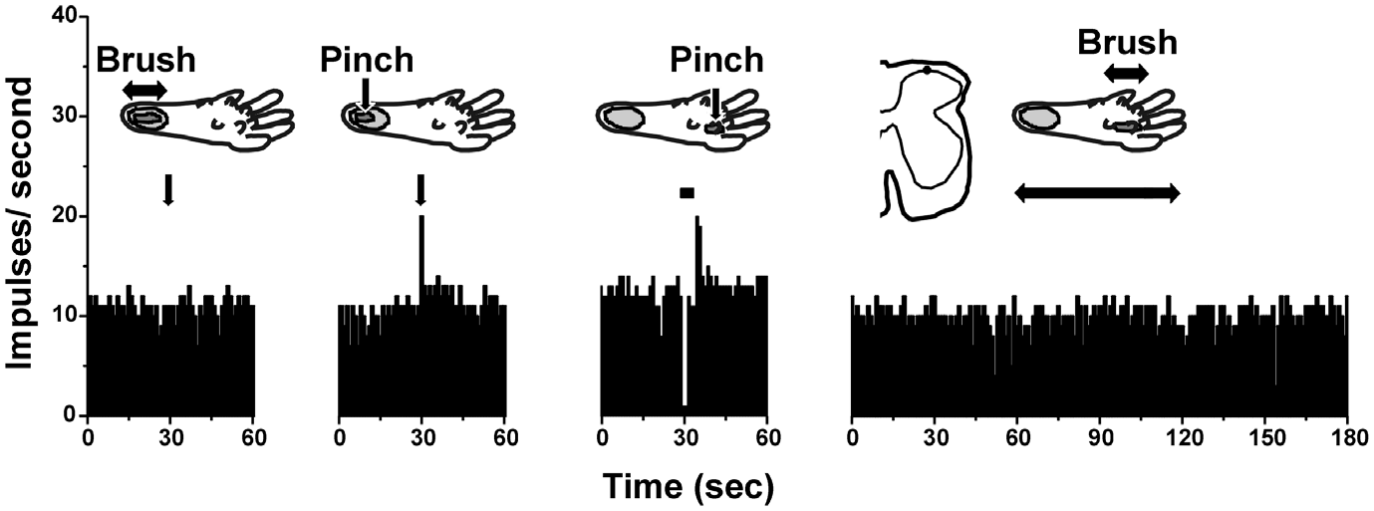
Pinch-evoked inhibition of spinal neuronal firing. PSTHs (bins: 1 sec) show responses of a superficial dorsal horn unit to brush and pinch stimuli delivered either within (2 left-hand PSTHs) or outside (2 right-hand PSTHs) the receptive field on the heel (gray area). The unit was excited by pinch but not brush within the receptive field, and was inhibited by pinch but not brush stimulus to outside receptive field.

Three units were tested with noxious pinch, which significantly inhibited ongoing firing (Fig. 2A, right PSTH; p<0.05, paired t-test). In one case it was possible to map the mechanosensitive receptive field on the heel (Fig. 4). The unit responded to pinching within the receptive field, but not innocuous brush either within or outside of the recepive field, and was classified as nociceptive-specific [12]. Pinching a footpad outside of the receptive field inhibited the unit.

### GABA and glycine antagonists reduce scratch-evoked inhibition

**Figure 5:**
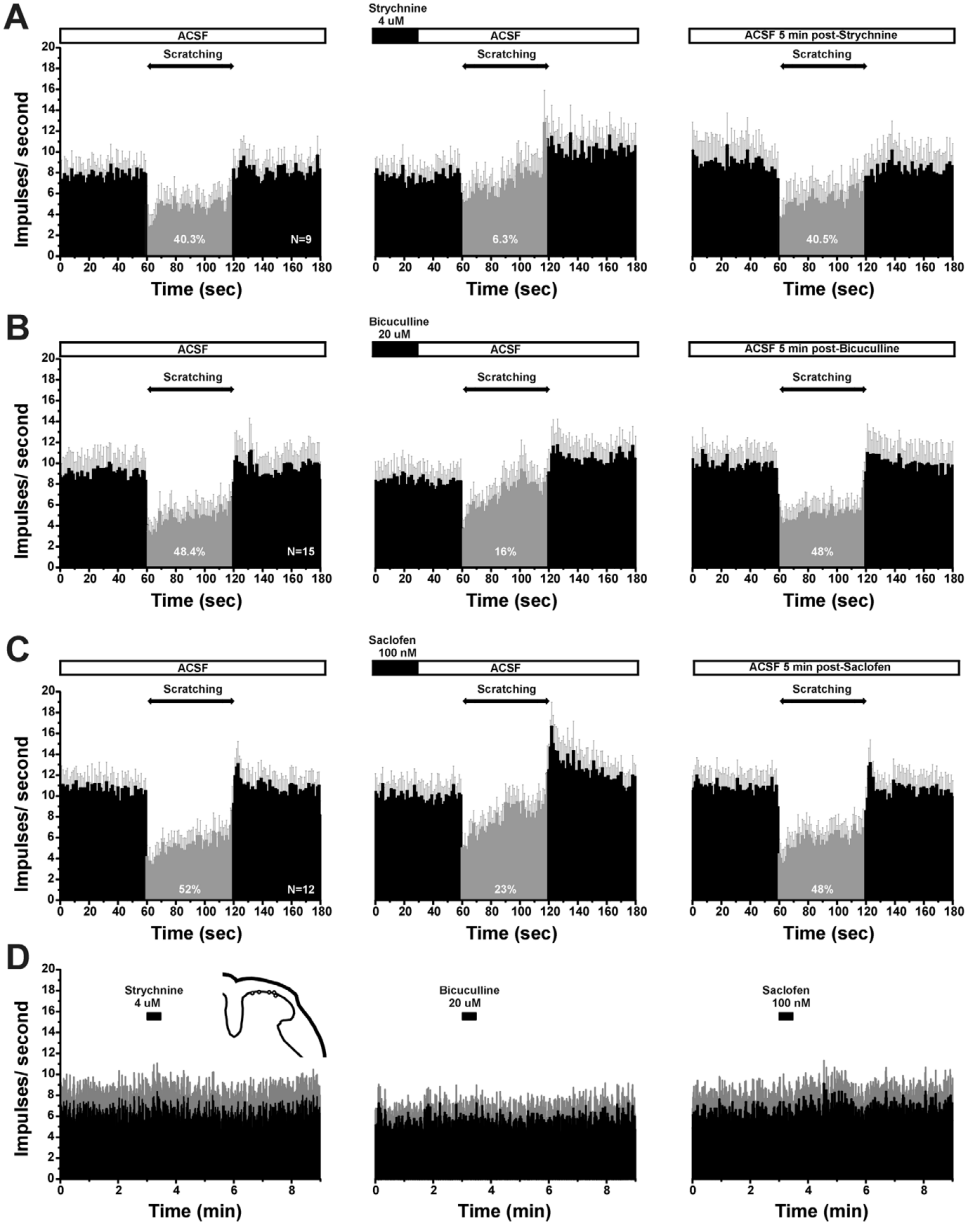
Glycine and GABA antagonists reduce scratch-evoked inhibition. A: PSTHs (bins: 1 sec) of averaged firing of 9 superficial dorsal horn units. Gray error bars: SEM. Left panel shows scratch-evoked depression of firing during ACSF superfusion. The gray portion of the PSTH highlights the scratch-evoked reduction in firing. Middle panel shows reduced inhibition following superfusion with glycine antagonist strychnine, and right panel shows recovery of scratch-evoked inhibition 5 min later. Percentages indicate the degree of suppression of firing during scratching, relative to pre-scratch baseline. B: Mean activity of 15 units before, during and after spinal superfusion with GABA_A_ antagonist bicuculline (format as in A). C: Mean activity of 12 units with GABA_B_ antagonist saclofen (format as in A, B). D: PSTHs (bins: 1 sec) of averaged firing of superficial dorsal horn units. Gray error bars: SEM. Left panel shows ongoing firing after strychnine superfusion (*n* = 8). Middle panel shows ongoing firing following superfusion with bicuculin (*n* = 8). Right panel shows ongoing firing following superfusion with saclofen (*n* = 8). None of the drugs affected ongoing firing.

We tested if antagonism of spinal glycine and GABA receptors affected scratch-evoked suppression of unit firing, using the glycine antagonist strychnine and antagonists of GABA_A_ (bicuculline) and GABA_B_ (saclofen) receptors. Overall, strychnine was more effective that the GABA antagonists in attenuating the scratch-evoked suppression of spontaneous firing. Fig. 1A shows an individual example in which strychnine reduced scratch-evoked inhibition of unit firing. Fig. 5A shows averaged data for 9 units tested with strychnine. Under control conditions (ACSF superfusion), average spike counts/60 sec were significantly (p<0.01) reduced (by 40.3%) during scratching, compared to the pre-scratch level of activity (Fig. 5A, left PSTH). One minute following spinal superfusion of strychnine, scratching no longer significantly affected firing, with the mean spike count during scratching being reduced by only 6.3% (Fig. 5A, middle PSTH). At this time point, the mean spike count/60 sec during scratching was significantly greater compared to that prior to strychnine (p<0.05, paired t-test). Five minutes later, scratching inhibited ongoing neuronal activity to the same degree as observed before application of strychnine (40.5%; Fig. 5A, right-hand PSTH).

The respective GABA_A_ and GABA_B_ antagonists bicuculline and saclofen also both significantly reduced scratch-evoked suppression of neuronal firing, but to a lesser degree compared to strychnine. One minute following bicuculline, the mean spike count/60 sec was reduced less during scratching (16%) than prior to bicuculline (48.4%) (Fig. 5B, left-hand and middle PSTHs). Accordingly, the mean spike count during scratching was significantly greater compared to the pre-bicuculline count (p<0.05). Five minutes later, scratch-evoked inhibition had recovered to the pre-bicuculline level (48%; Fig. 5B, right-hand PSTH). Similarly, 1 min following saclofen, scratching reduced spike counts by 23% compared to a 52% reduction prior to saclofen (Fig. 5C, left-hand and middle PSTHs). Mean spike counts during scratching were significantly greater 1 min after sacolfen compared to pre-saclofen (p<0.01). Five minutes later there was complete recovery of scratch-evoked inhibition (48%; Fig. 5C, right-hand PSTH). None of the antagonists administered alone had any significant effect on ongoing firing (Fig. 5D).

### Upper cervical blockade reduces scratch-evoked inhibition

**Figure 6:**
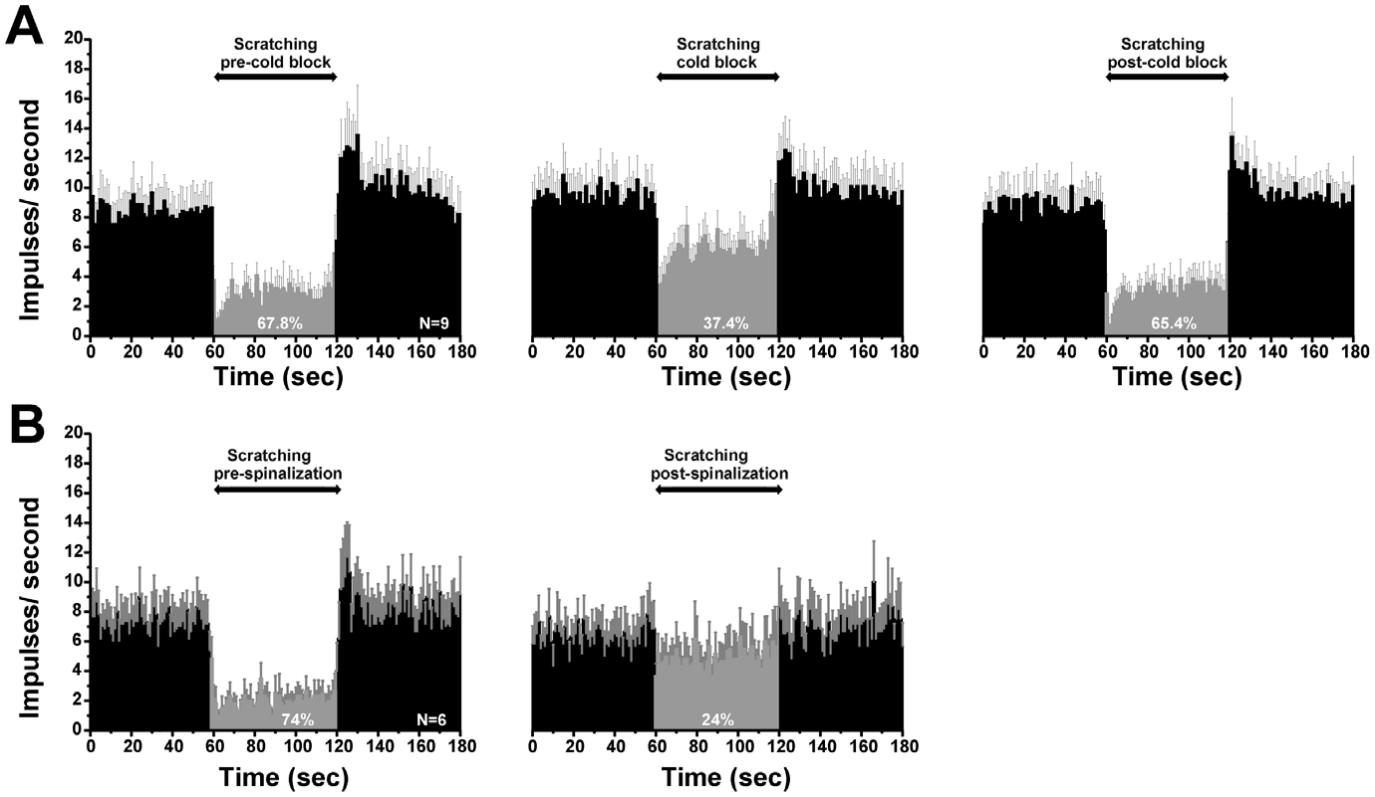
Disruption of upper cervical spinal cord reduces scratch-evoked inhibition. A: cold block. Left-hand PSTH shows averaged firing of 9 units before and during scratching under control conditions. Middle PSTH shows reduction in the degree of scratch-evoked inhibition during cold-block of upper cervical cord. Right-hand PSTH shows recovery of scratch-evoked inhibition after rewarming the cervical cord. B: Spinalization. Shown are mean PSTHs of 6 units from separate animals, before (lefthand PSTH) and after (right-hand PSTH) complete transection of the upper cervical spinal cord.

Does scratching exclusively inhibit neuronal activity at the spinal level, or does it activate a supraspinal loop? To address this question, we tested if scratch-evoked inhibition was reduced following reversible cold-block of the upper cervical spinal cord. The left-hand PSTH of Fig. 6A shows mean scratch-evoked suppression of spontaneous activity in 9 units. During cold-block of the upper cervical spinal cord (Fig. 6A, middle PSTH), the inhibitory effect of scratching was reduced by 30%. Scratching reduced the mean spike count to a lesser degree (37.4%) compared to the stronger inhibition before cold block (67.8%). The mean spike count during scratching was significantly greater (p<0.05, paired t-test) during cold block compared to pre-cold block. These data indicate that scratching activates ascending sensory pathways that engage a supraspinal system that descends to inhibit dorsal horn neuronal firing. Since scratch-evoked inhibition was only partially reduced during cold block, this suggests that scratching also activates segmental inhibitory networks to suppress dorsal horn firing. However, an alternative explanation is that scratch-evoked inhibition depends exclusively on a supraspinal loop, and that the cervical cold block only partially blocked spinal transmission. To explore this possibility, in a final series of experiments we tested the effect of complete transection of the upper cervical spinal cord on scratch-evoked inhibition. Fig. 6B shows that after transection, scratching was 50% less effective in reducing neuronal firing. After spinal transection, scratching still reduced neuronal firing (by 24%; Fig. 6B, right-hand PSTH) but to a lesser degree than before transection (74%; Fig. 6B, left-hand PSTH). That cold-block and complete transection of the upper cervical spinal cord only partially reduced scratch-evoked inhibition of ongoing spinal neuronal firing indicates the participation of both segmental and suprasegmental mechanisms.

## Discussion

We presently identified neurons in the superficial dorsal horn that exhibited high rates of spontaneous activity that we postulate is due to ongoing pruriceptive input from the chronic dry itchy skin. Of the units for which it was possible to accurately test mechanical sensitivity, the majority were mechanically insensitive and the rest were classified as WDR or NS. In our recent study of superficial dorsal horn units similarly recorded in hindpaw dry skin-treated animals, 24% of units tested were mechanically insensitive, 32% were WDR and 44% NS [12]. These proportions are very similar to those of pruritogen-responsive superficial dorsal horn neurons recorded in naïve animals [15]–[16], indicating that the dry skin treatment did not markedly alter the neurons' mechanosensory properties. Spontaneous firing was significantly attenuated by scratching or heating the skin. Moreover, those units tested responded to pruritogens, consistent with our previous report [12]. All of these observations are consistent with a role for these spinal neurons in the ascending transmission of itch and/or as interneurons in local scratch reflex circuits.

### Inhibition of itch signaling by counterstimuli

Scratching significantly suppressed ongoing firing of superficial dorsal horn neurons while innocuous brushing was ineffective. Noxious heat and pinch also suppressed neuronal firing. These data are consistent with human studies showing that noxious but not innocuous counterstimuli reduced experimentally-induced itch [1], [17]. Cutaneous scratching had state-dependent effects on monkey spinothalamic tract neurons, whereby the responses of such neurons to the pruritogens histamine and cowhage were inhibited, while responses of the same neurons to capsaicin were facilitated, by cutaneous scratching [3]. Cowhage (Mucuna pruriens) is a tropical legume with seed pod spicules containing mucunain, which acts at protease-activated receptors PAR-2 and -4 to induce itch [18]. We hypothesize that the presently-observed spontaneous firing of superficial dorsal horn neurons reflects an “itch” state driven by ongoing pruriceptive input from dry skin, and would be subject to state-dependent inhibition by scratching, pinch and noxious heat.

Skin cooling usually reduced or abolished experimental itch in humans [2], [19]–[22], although in one study cooling increased the latency but otherwise did not reduce the magnitude or duration of itch elicited by intradermal histamine [23]. Skin cooling significantly attenuated the responses of rat dorsal horn neurons to histamine [24]. In the present study, intense skin cooling excited most (5/6) units tested and inhibited one. We recently reported that a substantial proportion of pruritogen- (5-HT and PAR-2 agonist) responsive neurons in mouse superficial dorsal horn were excited by skin cooling [15]–[16], We speculate that cooling provides both excitatory and inhibitory inputs to spinal neurons, with the net effect dependent on whether the excitatory (e.g., Fig. 3B) or inhibitory (e.g., Fig. 3A) input dominates.

### Glycine and GABA involvement in scratch-evoked inhibition

The present data support the hypothesis that scratching inhibits dry skin itch-related spontaneous firing of dorsal horn neurons via the intraspinal release of glycine and GABA. The glycine antagonist strychnine, and the GABA_A_ and GABA_B_ antagonists bicuculline and saclofen, respectively, all significantly attenuated scratch-evoked inhibition, with strychnine being the most effective. Both glycine and GABA have long been implicated in the inhibition of spinal nociceptive transmission [25]. Glycine and GABA are colocalized in spinal presynaptic terminals, with glycinergic inhibition of superficial nociceptive neurons dominating in adults [26]. It was recently reported that mice lacking Bhlhb5, a transcription factor expressed in the spinal cord dorsal horn during development, developed self-inflicted skin lesions and exhibited enhanced pruritogen-evoked scratching behavior [27]. This was associated with a selective loss of inhibitory spinal interneurons particularly in the superficial dorsal horn [27]. It was suggested that such interneurons mediate the inhibition of itch-signaling neurons elicited by scratching and other noxious counterstimuli, and that loss of these interneurons results in disinhibition of itch transmission [27]. Mice lacking the vesicular glutamate transporter VGLUT2, which is co-expressed in Nav1.8-expressing primary afferent fibers, also exhibited enhanced spontaneous and pruritogen-evoked scratching, as well as a reduction in nocifensive behavior [28]-[29]. This suggests that nociceptors normally release glutamate from their intraspinal terminals to excite inhibitory interneurons that suppress itch-signaling neurons. The loss of the inhibitory interneurons, or loss of excitatory nociceptive input, disinhibits spinal itch transmission to result in a phenotype of chronic itch manifested by spontaneous scratching and hyperknesis (enhanced itch). Our present data indicate a crucial role for glycine and GABA as neurotransmitters mediating the inhibition of itch-signaling spinal neurons elicited by noxious counterstimuli.

Spinal superfusion of the glycine and GABA antagonists alone had no effect on spontaneous firing (Fig. 5D), indicating an absence of glycinergic or GABAergic tone. We speculate that development of the dry skin condition is accompanied by a decrease in inhibitory interneuronal function which may contribute to the increase in spontaneous scratching behavior.

### Supraspinal and spinal modulation of itch transmission

Cold-block or complete transection of the upper cervical spinal cord reduced the inhibitory effect of scratching by 30 and 50%, respectively, implying that scratch-evoked inhibition is mediated partially via activation of supraspinal neurons that, in turn, engage descending pathways to result in spinal release of glycine and GABA. Following upper cervical spinal transection, scratching was still able to inhibit neuronal firing, implying the participation of a segmentally-organized inhibitory network in addition to the supraspinal mechanism. The segmental inhibitory circuit may involve inhibitory interneurons that are activated by nociceptive glutamatergic afferents, as discussed above. The supraspinal circuit is unknown, but speculatively may involve neurons in the rostral ventromedial medulla with descending projections to the spinal cord involved in modulation of nociceptive transmission [30].

**Figure 7:**
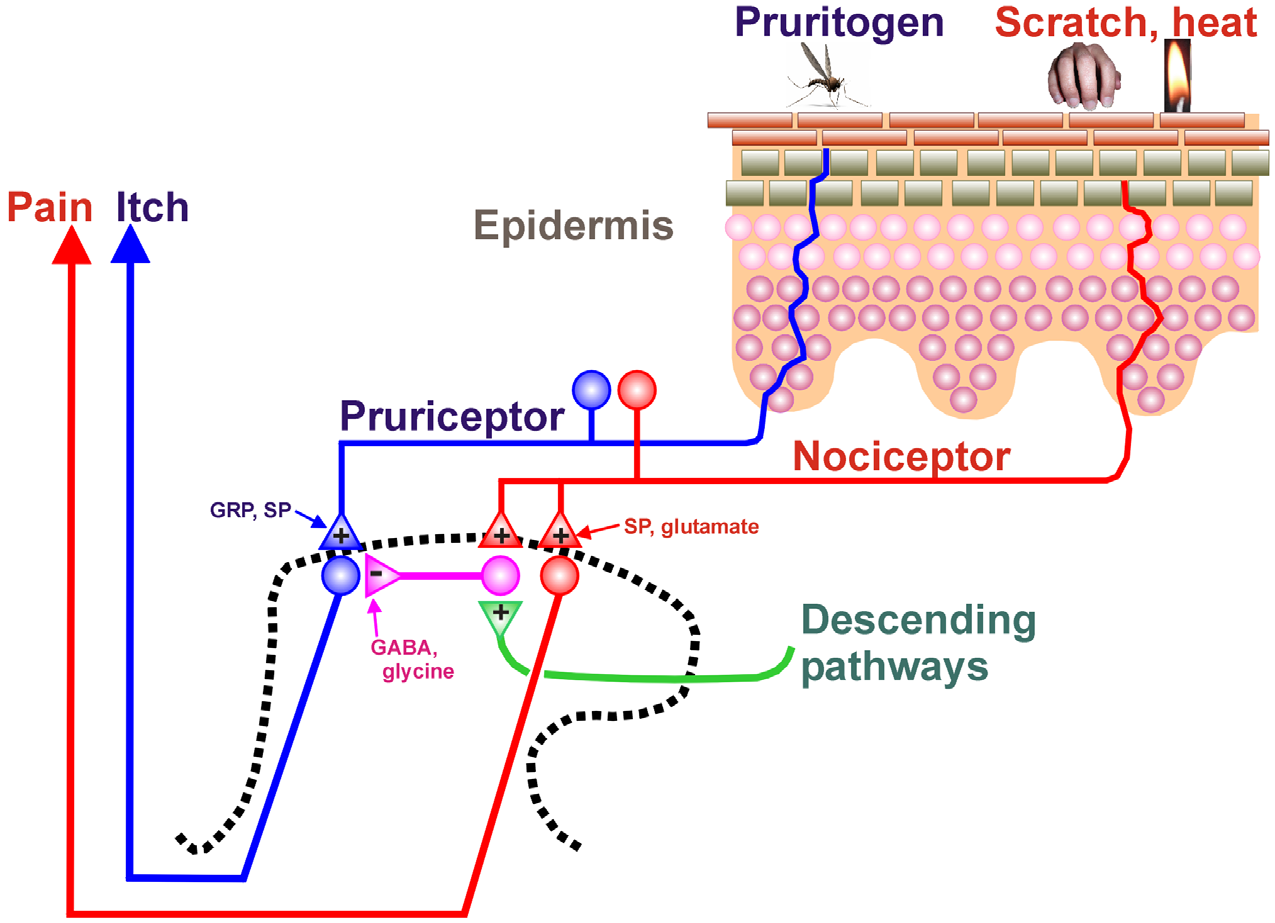
Schematic diagram showing epidermis (upper right) and an outline of the spinal dorsal horn (dashed line, lower left). +: excitatory synapse; -: inhibitory synapse. See text for further discussion.

A model for scratch-evoked inhibition is provided in Fig. 7. Itch-signaling neurons in the superficial dorsal horn receive input from primary afferent “pruriceptors” that are activated by pruritic agents. These include mechanically-sensitive C-fiber polymodal nociceptors that respond to cowhage [31]–[32], mechanically-insensitive C-fiber nociceptors that respond to histamine [31], [33], and fibers expressing mas-related G-protein-coupled receptors (mrgprs) [34], one of which (mrgprA3) responds to the anti-malarial drug chloroquine that commonly induces itch [35]. The intraspinal terminals of pruriceptors are thought to release gastrin releasing peptide (GRP) and/or substance P as neuropeptide transmitters to excite postsynaptic neurons expressing GRP and/or NK-1 receptors. This is based on evidence that mutant mice lacking GRP receptors [36], or neurotoxic destruction of neurons expressing GRP [37] or NK-1 receptors [38], significantly attenuates or abolishes pruritogen-evoked scratching behavior. The precise location of pruriceptive nerve endings in the epidermis is not clear; peptidergic afferents penetrate into mid-epidermal cell layers and some mrgpr-expressing afferent endings are more superficial [39] (Fig. 7, blue).

Scratching the skin presumably activates mechanically-sensitive C-fiber polymodal nociceptors with primary afferent projections to the superficial dorsal horn of the spinal cord (Fig. 7, red). These afferents are postulated to express Nav1.8 and VGLUT2, and release glutamate to excite inhibitory interneurons in the superficial dorsal horn (Fig. 7, magenta). The inhibitory interneurons use glycine and GABA as neurotransmitters to inhibit pruritogen-responsive neurons. Scratching also excites ascending nociceptive neurons that project to supraspinal structures that, in turn, directly or indirectly connect with descending modulatory pathways that are proposed to excite the same spinal glycinergic/GABAergic inhibitory interneurons (Fig. 7, green). In this regard, it was recently reported that descending noradrenergic, but not serotonergic, pathways exert tonic inhibition on itch signaling in the spinal cord [40]. It will be of considerable future interest to identify the supraspinal structures involved in descending inhibition of itch-signaling spinal neurons, and to determine if they overlap with known pain-modulatory pathways in the midbrain periaqueductal gray and rostral ventromedial medulla.

The present identification of a major role for spinal glycine and GABA in the mediation of scratch-evoked inhibition of pruriceptive neurons provides new targets for development of antipruritic treatments that take advantage of segmental and suprasegmental itch-inhibitory networks.

## References

[1] Ward L, Wright E, McMahon SB (1996). A comparison of the effects of noxious and innocuous counterstimuli on experimentally induced itch and pain.. Pain.

[2] Murray FS, Weaver MM (1975). Effects of ipsilateral and contralateral counterirritation on experimentally produced itch in human beings.. J Comp Physiol Psychol.

[3] Davidson S, Zhang X, Khasabov SG, Simone DA, Giesler GJ (2009). Relief of itch by scratching: state-dependent inhibition of primate spinothalamic tract neurons.. Nat Neurosci.

[4] White JC, Sweet WH (1969). Pain and the Neurosurgeon: A Forty Year Experience..

[5] Akiyama T, Merrill AW, Zanotto K, Carstens MI, Carstens E (2009). Scratching behavior and Fos expression in superficial dorsal horn elicited by protease-activated receptor agonists and other itch mediators in mice.. J Pharmacol Exp Ther.

[6] Carstens E, Castro-Lopes J (2009). Neurobiology of itch and pain: scratching for answers.. Seattle.

[7] Tsujii K, Andoh T, Lee JB, Kuraishi Y (2008). Activation of proteinase-activated receptors induces itch-associated response through histamine-dependent and -independent pathways in mice.. J Pharmacol Sci.

[8] Miyamoto T, Nojima H, Shinkado T, Nakahashi T, Kuraishi Y (2002). Itch-associated response induced by experimental dry skin in mice.. Jpn J Pharmacol.

[9] Nojima H, Cuellar JM, Simons CT, Carstens MI, Carstens E (2004). Spinal c-fos expression associated with spontaneous biting in a mouse model of dry skin pruritus.. Neurosci Lett.

[10] Akiyama T, Carstens MI, Carstens E (2010). Spontaneous itch in the absence of hyperalgesia in a mouse hindpaw dry skin model.. Neurosci Lett.

[11] Yamaguchi T, Nagasawa T, Satoh M, Kuraishi Y (1999). Itch-associated response induced by intradermal serotonin through 5-HT2 receptors in mice.. Neurosci Res.

[12] Akiyama T, Carstens MI, Carstens E (2011). Enhanced responses of lumbar superficial dorsal horn neurons to intradermal PAR-2 agonist but not histamine in a mouse hindpaw dry skin itch model.. J Neurophysiol.

[13] Narikawa K, Furue H, Kumamoto E, Yoshimura M (2000). In vivo patch-clamp analysis of IPSCs evoked in rat substantia gelatinosa neurons by cutaneous mechanical stimulation.. J Neurophysiol.

[14] Lomber SG, Payne BR, Horel JA (1999). The cryoloop: an adaptable reversible cooling deactivation method for behavioral or electrophysiological assessment of neural function.. J Neurosci Methods.

[15] Akiyama T, Merrill AW, Carstens MI, Carstens E (2009). Activation of superficial dorsal horn neurons in the mouse by a PAR-2 agonist and 5-HT: potential role in itch.. J Neurosci.

[16] Akiyama T, Carstens MI, Carstens E (2009). Excitation of mouse superficial dorsal horn neurons by histamine and/or PAR-2 agonist: potential role in itch.. J Neurophysiol.

[17] Bickford RG (1938). Experiments in relation to the itch sensation—its peripheral mechanism and central pathways.. Clin Sci.

[18] Reddy VB, Iuga AO, Shimada SG, LaMotte RH, Lerner EA (2008). Cowhage-evoked itch is mediated by a novel cysteine protease: a ligand of protease-activated receptors.. J Neurosci.

[19] Bromm B, Scharein E, Darsow U, Ring J (1995). Effects of menthol and cold on histamine-induced itch and skin reactions in man.. Neurosci Lett.

[20] Fruhstorfer H, Hermanns M, Latzke L (1986). The effects of thermal stimulation on clinical and experimental itch.. Pain.

[21] Halkier-Sorensen L, Thestrup-Pedersen K (1989). The relevance of low skin temperature inhibiting histamine-induced itch to the location of contact urticarial symptoms in the fish processing industry.. Contact Dermatitis.

[22] Melton FM, Shelley WB (1950). The effect of topical antipruritic therapy on experimentally induced pruritus in man.. J Invest Dermatol.

[23] Simone DA, Alreja M, LaMotte RH (1991). Psychophysical studies of the itch sensation and itchy skin (“alloknesis”) produced by intracutaneous injection of histamine.. Somatosens Mot Res.

[24] Jinks SL, Carstens E (1998). Spinal NMDA receptor involvement in expansion of dorsal horn neuronal receptive field area produced by intracutaneous histamine.. J Neurophysiol.

[25] Zeilhofer HU (2005). The glycinergic control of spinal pain processing.. Cell Mol Life Sci.

[26] Chery N, de Koninck Y (1999). Junctional versus extrajunctional glycine and GABA(A) receptor-mediated IPSCs in identified lamina I neurons of the adult rat spinal cord.. J Neurosci.

[27] Ross SE, Mardinly AR, McCord AE, Zurawski J, Cohen S (2010). Loss of inhibitory interneurons in the dorsal spinal cord and elevated itch in Bhlhb5 mutant mice.. Neuron.

[28] Liu Y, Abdel Samad O, Zhang L, Duan B, Tong Q (2010). VGLUT2-dependent glutamate release from nociceptors is required to sense pain and suppress itch.. Neuron.

[29] Lagerstrom MC, Rogoz K, Abrahamsen B, Persson E, Reinius B (2010). VGLUT2-dependent sensory neurons in the TRPV1 population regulate pain and itch.. Neuron.

[30] Ossipov MH, Dussor GO, Porreca F (2010). Central modulation of pain.. J Clin Invest.

[31] Namer B, Carr R, Johanek LM, Schmelz M, Handwerker HO (2008). Separate peripheral pathways for pruritus in man.. J Neurophysiol.

[32] Johanek LM, Meyer RA, Friedman RM, Greenquist KW, Shim B (2008). A role for polymodal C-fiber afferents in nonhistaminergic itch.. J Neurosci.

[33] Schmelz M, Schmidt R, Bickel A, Handwerker HO, Torebjork HE (1997). Specific C-receptors for itch in human skin.. J Neurosci.

[34] Dong X, Han S, Zylka MJ, Simon MI, Anderson DJ (2001). A diverse family of GPCRs expressed in specific subsets of nociceptive sensory neurons.. Cell.

[35] Liu Q, Tang Z, Surdenikova L, Kim S, Patel KN (2009). Sensory neuron-specific GPCR Mrgprs are itch receptors mediating chloroquine-induced pruritus.. Cell.

[36] Sun YG, Chen ZF (2007). A gastrin-releasing peptide receptor mediates the itch sensation in the spinal cord.. Nature.

[37] Sun YG, Zhao ZQ, Meng XL, Yin J, Liu XY (2009). Cellular basis of itch sensation.. Science.

[38] Carstens EE, Carstens MI, Simons CT, Jinks SL (2010). Dorsal horn neurons expressing NK-1 receptors mediate scratching in rats.. Neuroreport.

[39] Dussor G, Koerber HR, Oaklander AL, Rice FL, Molliver DC (2009). Nucleotide signaling and cutaneous mechanisms of pain transduction.. Brain Res Rev.

[40] Gotoh Y, Omori Y, Andoh T, Kuraishi Y (2011). Tonic inhibition of allergic itch signaling by the descending noradrenergic system in mice.. J Pharmacol Sci.

